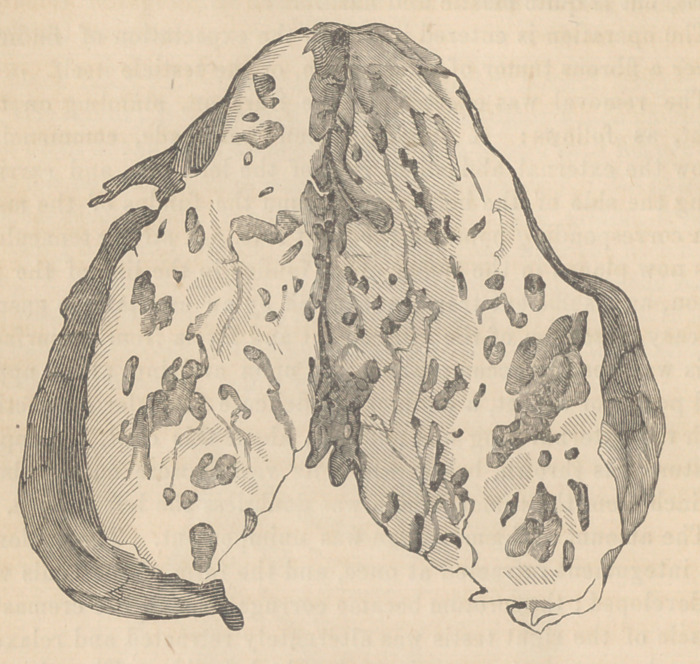# Pennsylvania College, Ninth below Locust Street

**Published:** 1853-03

**Authors:** W. H. Gobrecht


### CLINICAL REPORTS.

*Pennsylvania College, Ninth below Locust street. Service of* Professor GILBERT.

Reported by W. H. GOBRECHT, M. D.

*Jan. 22d. Congenital Varus.*—Robert C——, (Case LXIII.) Dressings re-applied. A gaiter was ordered to be made upon the steel sole, to lace in front, and to have a good catch on the heel, so that it may be easily applied and effectually worn.

*Spinal Irritation.*—Robert C——, (Case LXVII.) This patient has greatly improved since the introduction of the seton, having had but two paroxysms, and those of a lighter character than usual, since his last visit. There seems to be, however, some slight epileptic complication, and as Prof. Gilbert has found great benefit to result from the use of Veratria in nervous affections, especially in neuralgia, given until the production of tingling in the fingers and nausea, the Iodide of Potassium is discontinued, and the following prescription ordered:

℞ Veratriæ gr. ij.
Ft. in pil. No. xvi.
S. one thrice daily.

When the patient feels that a paroxysm is approaching, an additional pill is to be taken.

Feb. 5th. Has had no decided spasm since his last appearance, and but slight tremors twice only; he walks better, looks better and has now no headache. He is directed to take a Veratria pill *four* times, instead of thrice daily, and an additional pill if any premonitory tremors are noticed; but if much nausea results, the medicine should be suspended.

*Strabismus.*—James C——, (Case LXIX.) Some pain being set up in the eye after the operation, the patient was purged and cold water applied with relief.

The parallelism of the globes is perfect.

Case LXXIII. *Results of extensive injuries.*—James F——, aged 11 years, whilst flying a kite on a three storied house, during the last summer, fell to the pavement and incurred a dislocation of the right radius and ulna, backward, at the elbow-joint, with fracture of the coronoid process, also a compound fracture of the same bones below the joint, with anterior protrusion; there was a luxation of the left wrist backward, with compound fracture of radius and ulna near the elbow joint. In the consideration of the question of amputation, the almost utter helplessness of the patient which would follow the removal of both superior extremities, or if one only should be sacrificed, the serious impediment to success in the treatment of its stump from the condition of the other, determined an attempt to save both, which resulted thus: The right elbow is perfect, the radius is a very little impeded in rotation, and the length of the forearm undiminished. In regard to the left extremity, about an inch of the shaft of the radius was lost during treatment; there is partial rigidity of the fore and mid fingers, but good use of the thumb, ring and little fingers, so that the patient can grasp with some facility, which doubtless will increase.

The treatment pursued after reduction and dressing the wounds, was by means of wooden splints padded with cotton. These were subsequently cut so as to afford access to the suppurating wounds, to which poultices were applied. Constitutional treatment of a rigidly antiphlogistic character became necessary during the first two weeks, after which there was no sympathetic fever.

Case LXXIV.—*Tumor on the Eye.*—Olivia C——, aged 49. Married. This tumor, about the size of a common white bean, is situated beneath the conjunctiva of the right eye, at the outer margin of the cornea, and in the antero-posterior axis of the globe; it seems to be firmly attached to the tissues beneath, and perhaps involves the cornea, which it partially overlies. Its appearance is that of a fibrous growth, although we may perhaps find it to be of a heterologous character. Its history is briefly this: When a child, the patient was struck and injured slightly in the spot now occupied by the tumor, with a stick, which caused no noticeable unpleasant result. About fifteen years since, the adventitious growth was first perceived, and gradually increased. Five years ago, Dr. Geo. M'Clellan removed it; the remaining ulcer cicatrized well, and nothing further was feared; however, it has recently reappeared, and for the last six months has grown more rapidly than at any previous time, causing, by its presence much irritation and frequent attacks of inflammation.

The patient is informed that its removal endangers the integrity of the eye, which may be pierced during the operation; besides which it may return, even if the procedure is successful. Nevertheless, if the tumor is allowed to remain, destruction of the organ is inevitable.

The patient being seated, a fine tenaculum was passed into the adventitious mass, but cut out at once; it was then shaved off by a sharp scalpel, and Nitrate of Silver applied to the cut surface.

The tumor is doubtless a fungous degeneration of a fibrous growth, having a deep-seated base, and we fear may eventually involve the entire eye, demanding its extirpation.

26th. Eye somewhat irritated, but no unpleasant symptoms. Nitrate of Silver reapplied.

*Jan 29th.* CASE LXXV. *Amaurosis.*—Albion D. S——, sanguineous temperament, aged 29, oyster dredger, was seized, three weeks since, with pain in the temples, and glimmering of vision, which became at first lightly and then more densely foggy, until, although able to see light, he has become unable to distinguish objects, the left eye failing first. His appetite is good and bowels regular.

Loss of vision may result from several causes, as from cataract, an opacity of the crystalline lens; from glaucoma, a disease of the vitreous humor; or from amaurosis, an affection of the retina.

In this case however, the pupil is dilated and is of a natural color, hence neither cataract nor glaucoma exist; no acute symptoms have been present, indicating retinitis; and no general paralysis indicating nervous disease, is found; thus, doubtless, amaurosis has resulted from mere *congestion* of the vessels of the retina. Moreover, there is a little motion in the iris, which is not usually the case in confirmed amaurosis.

Venesection was ordered, the flow of blood to be continued almost to fainting. Then

℞. Hydrarg. chlo. mit. gr. x., to be followed in two hours with

Magnesiæ sulphat. ℥ss.

Sennæ ʒj.

Aq. bullient. f. ℥iv.

S. To be taken at one dose.

After the operation of the cathartic, to take the following:

℞. Hydr. chlo. mit. gr. xvi.
Potass. nitrat. ʒij.
Ant. et Potass. Tart. gr. ij.
M. ft. in chart No. xvi.
S. one thrice daily.

He was also directed to apply to the eye externally by saturated cloths:

℞. Plumbi Acetat. ʒj.

Aquæ fontis ℥viij.

M. ft. in lot.

Feb. 5th. As we see him to-day, vision has greatly improved in the left eye, but some iritis exists in the right, for which he is directed to renew the calomel, nitre and antimony, and take as before directed; besides which a seton is passed in the back of his neck as a powerful counter-irritant. Diet moderate, and occasional purging.

CASE LXXVI.—*Cystic-sarcoma of the left testicle, congenital,* in Wm. Henry B——, aged 3 years. This tumor which existed at the birth of the child as above stated, has grown with his growth, but has not increased in size, relatively to the body. Its external appearance is well represented in the cut, (by Gihon, from a drawing by Mr. J. Wilson,) as extending from near the abdominal ring of the left side, very nearly to the knee, filling up the entire scrotum and drawing down the integument of the abdomen to such an extent as to obliterate any appearance of a penis, the extremity of the prepuce only being visible.

It corresponds very well to the description of tumors in this situation which occur in warm climates.

It is not hydrocele, since there is neither translucency nor fluctuation. It is not enterocele, since there is no well marked connexion with the abdominal ring, and it is too firm for such a tumor. It is not epiplocele, since it is not doughy and very unequal, but is quite elastic and hard.

The operation is entered into with the expectation of finding, either a fibrous tumor of the scrotum, or the testicle itself.

The removal was effected by the Surgeon, standing on the right, as follows: A single incision was made, commencing below the external abdominal ring of the left side, and carried along the side of the body and around the fundus of the mass to a corresponding point on the right side. A strong tenaculum was now placed in the tumor at its fundus in the line of the incision, and sufficient traction exerted by an assistant to ensure an easy dissection of the integument and fascia from its surface. This was readily accomplished, but upon arriving at its upper and posterior aspect, a distinct but delicate funicular connection with the external ring was noticed; about this cord, high up, a ligature was thrown, below which its vessels were cut at about an inch from the tumor, which was doubtless the left testicle.

The amount of hæmorrhage was unimportant. The abdominal integument retracted at once, and the form of the penis was re-developed; the scrotum became corrugated, and the cremaster muscle of the right testis was alternately retracted and relaxed, no such movements occurring upon the left side. The edges of the incision, (now very much diminished in length,) were brought together by four twisted sutures, and the scrotum supported by strips of adhesive plaster.

The case was taken charge of by Dr. Fussell of this city, who also assisted in the operation.

Feb. 2d. Symptoms of local inflammation, with symptomatic fever, were fully developed on the second day after the operation, requiring the application of ten leeches, and the internal exhibition of Hyd. Chlor. Mit., followed by Magnes. Sulph. Subsequently,

℞. Potass. Nitrat., ʒss.

Ant. et Potass. Tart. gr. i.

Aquæ font. f℥iv.

S. A tea spoonful every two hours.

This treatment reduced the local and general symptoms in a few days; since which they have entirely subsided.

The tumor, as here shown (in a cut by Gihon, from a drawing by Mr. J. Wilson,)

is $5\frac{5}{8}$ inches in length. It is enclosed in a firm fibrous structure, which has a serous covering. Several vessels enter its upper and posterior aspect.

On dividing the mass longitudinally, it is found composed of tortuous crypts of variable size filled with a transparent glutinous fluid, separated by fibrous bands, dotted here and there with black; from one cyst larger than the rest at the lower angle, some apparently strumous flocculi were discharged. The tumor is, in all probability, a *cystic sarcoma* of the testis, which is rare, a very few such cases being reported. The cysts are enlargements of the tubuli semeniferi, quite analogous to the condition which exists in cystic sarcomata of the breast. The following is a microscopic analysis of its contents, by Prof. F. G. Smith, of this Institution:

Calcareous deposits in the tunica vaginalis testis.

Cholesterine and fat cells.

Pus globules and glandular tissue.

Melanotic cells.

Colloid matter, no cancer cells, but large inorganic globules, granulous matter.

We must conclude, therefore, that whilst there is no *positive* malignancy in this tumor, that it is proper to follow the case closely, and observe if there be any further developements.

CASE LXXVII. *Porrigo favosa.*—Christiana S——, aged 19 months. This is the most common form of scald head. It consists of favous pustules, resulting in scabs, and is contagious.

The scabs are circular and cup-shaped; they enlarge gradually, and multiply until a great part of, or the whole scalp, may become involved; the contiguous cup-shaped depressions presenting the appearance of a honey-comb, from which resemblance the affection has received a part of its name, *favosa.*

This case is one of moderate severity.

It was directed that the scalp should be well cleansed with soap and water, by means of a fine sponge, and that Tar ointment should be applied morning and evening.

The following was prescribed for internal use:

℞. Potassii Iodidi, gr. vi.

Iodinii, gr. iii.

Aquæ fontis, f℥i.

S. 15 drops thrice daily.

*Feb. 2d. Sinus in Left Cheek.*—Delia M——, (Case LXVI.) The inflammation and swelling seem here to continue unusually long, so that, in all probability, some portion of a decayed fang still remains. On examination of the jaw, such a fang was found, and removed.

CASE LXXVIII. *Contusion resulting in Abscess.*—Thomas C——, aged 40. This patient, whilst running, about two weeks since, fell upon a curb stone, severely bruising his right elbow and arm. This injury has resulted in an abscess on the outside and back of the forearm, just below the elbow. Upon opening this abscess by a straight bistoury, in its most prominent part, as far from the joint as possible, pus of a strumous character was evacuated. Poultice ordered.

Case LXXIX. *Cataract, left eye, formative stage.*—Mr. W——, aged 55. The sight of the left eye in this person began to fail some eight months back, since which time the obstruction to vision has gradually increased. At present he can see that persons are before him, although he cannot distinguish them. In cloudy weather his vision is by far the best, (this is one of the surest diagnostic marks of cataract.) The pupil is of normal size, and presents a nebulous appearance. No operation would be justifiable at present.

Ordered purgatives and moderate diet, with protection of the part from cold.

Case LXXX. *Fracture of left clavicle.*—George McC——, aged 20. The fracture occurred seven weeks ago, just above the coracoid process of the scapula, and as no very great displacement results from such an injury at this point, the patient, unalarmed, did not apply for surgical aid until three weeks after the accident, when it seems that the ordinary treatment was pursued. The case is presented for the observation of the symptoms and results of this peculiar fracture. From the manner in which the clavicle is bound to the coracoid process, the fragments were but slightly displaced, and the patient, supposing a contusion only, was not startled until subsequent swelling came on from formation of callus. The injured clavicle is found, on measuring from the centre of the sternum to its acromial extremity, to be a half inch shorter than its fellow; the fractured ends do not perceptibly project upward, a mass of callus being found surrounding them.

*Double Cataract.* Margaret G——, (Case LXXII.)—In this case inflammation set in about 36 hours after the operation, for which she was bled from the arm to approaching syncope, after which a brisk purge was given, which operated well. The inflammation not yielding entirely, and becoming aggravated, on the ensuing day twenty leeches were applied to the temporal region of the same side, and the following powders ordered:

℞. Hyd. chlor. Mit. gr. xvj.
Potass. Nitrat. ʒij.
Ant. et Potass. Tart. gr. ij.

M. ft. in chart. No. xvj. S. One every four hours. These were continued until slight constitutional effects were had. The case resulted in a perfect cure.

CASE LXXXI. *Chronic Erythema.*—Charles M——, aged 65, a turner, always had good health, has chronic erythema of the dorsum of left foot and anterior part of leg, extending from the toes to just below the knee, with subcutaneous effusion.

The following ointment was ordered:

℞. Plumbi. Acetat. ʒij.

Cerat. Simp. ℥ij.

M. ft. ungt. S. To be applied on a cloth, evenly spread, and retained by a bandage from the toes up.

CASE LXXXII. *Orchitis.*—Charles H——, aged 27. Has subacute inflammation of the right testis, arising from cold, he was ordered:

℞. Hydrarg. Chlo. Mit. gr. v.

To be followed in two hours by:

℞. Magnesiæ Sulphat. ℥j.

And adhesive strips were rather firmly applied to the affected testicle in the presence of the class, in the manner ordinarily directed.

## Figures and Tables

**Figure f1:**
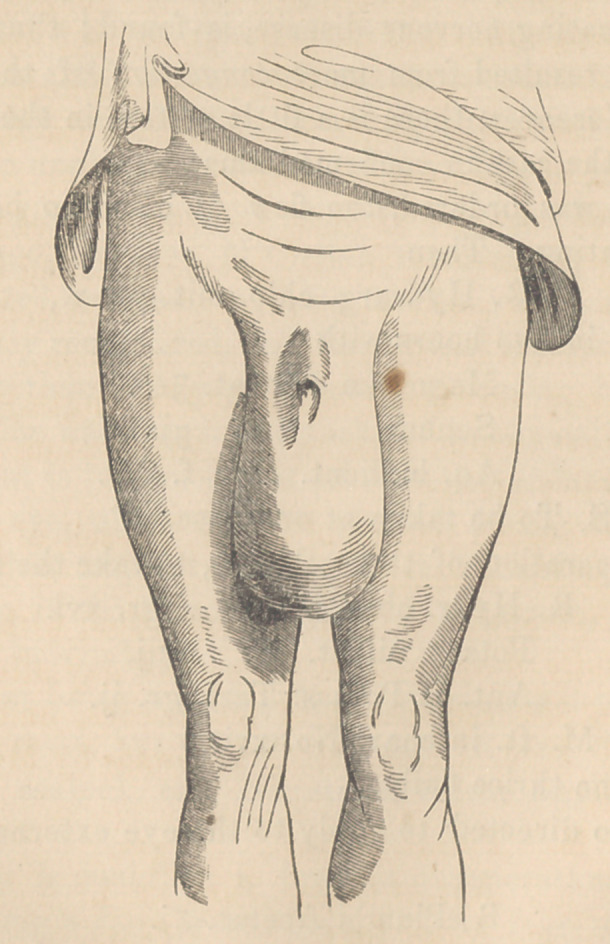


**Figure f2:**